# Association between in-hospital antibiotic use and long-term outcomes in critically ill patients

**DOI:** 10.1017/ash.2025.10054

**Published:** 2025-06-23

**Authors:** Parker Burrows, Ruth-Ann Brown, Abigail Samuelsen, Anthony S. Bonavia

**Affiliations:** 1 Department of Anesthesiology and Perioperative Medicine, Penn State Milton S Hershey Medical Center, Hershey, PA 17036, USA; 2 Institut de Genetique et de Biologie Moleculaire et Cellulaire, Cedex, France; 3 Critical Illness and Sepsis Research Center (CISRC), Penn State College of Medicine, Hershey, PA, USA

## Abstract

**Objective:**

To assess whether antibiotic duration (AD) and one-year antibiotic-free days (AFD) are associated with key in-hospital and post-discharge outcomes among critically ill adults.

**Design:**

Prospective observational study.

**Setting:**

611-bed, quaternary care academic medical center in the United States.

**Patients:**

126 critically ill adults (mean age 68.1 ± 15.6 yr, 51.6% male, median APACHE II score 20.5 [IQR 15–25]); 71.4% met sepsis criteria.

**Methods:**

Secondary infection was defined as ≥3 consecutive antibiotic days within a year after the index sepsis admission. Multivariate analyses adjusted for age, APACHE II score, BMI, and glucocorticosteroid dose. Time-to-event analysis employed Cox proportional hazards modeling; cumulative infection burden was assessed via nonparametric tests using normalized antibiotic exposure (AD as a proportion of days alive).

**Results:**

Within 30 days, longer AD correlated with increased hospital stay; each additional antibiotic day added ∼0.93 hospital days (*P* < 0.001) in adjusted linear regression. AD did not predict one-year mortality (OR 1.01, *P* = 0.739) or readmission (OR 1.01, *P* = 0.771). Normalized antibiotic exposure significantly differed by cumulative secondary infection episodes (*P* = 0.0033), with higher exposure among patients experiencing two or more secondary infections (*P* = 0.026 and *P* = 0.036, respectively). Cox regression showed a significant association between AD and time to first secondary infection (HR 1.10, 95% CI: 1.04–1.15, *P* = 0.001), indicating that longer AD predisposed to secondary infection or recurrent antibiotic use.

**Conclusions:**

Extended AD, in critically ill patients, prolongs hospitalization without reducing mortality or readmission rates. These findings highlight the importance of robust antibiotic stewardship practices, where shorter, targeted regimens may minimize unintended complications.

## Introduction

Sepsis remains a major global cause of morbidity and mortality, yet its diagnosis is inherently clinical.^1^ Current guidelines rely on the Sequential Organ Failure Assessment (SOFA) score^2^ to identify sepsis-related organ dysfunction, though no definitive biomarker exists. This diagnostic uncertainty drives empiric, broad-spectrum antibiotic use due to demonstrated survival benefits of early treatment.^3–7^ However, empirically initiated antibiotics are frequently continued beyond clinical necessity, increasing the risk of microbiome disruption and secondary infections.^8–11^

Given these concerns, we hypothesized that prolonged antibiotic exposure would increase secondary infection risk and hospital resource utilization without mortality benefit. We operationalized secondary infection as receipt of ≥3 consecutive antibiotic days post-index admission, differentiating empiric short-course therapy from treatment of clinically suspected infections.^12,13^ We do not presume that this cutoff defines an optimal AD for any given pathogen; rather, it serves as a practical tool for discerning short-term empiric treatment from courses intended for active infection management. By evaluating long-term outcomes—including secondary infections, mortality, and readmissions—this study informs best practices around antibiotic stewardship in intensive care units (ICUs).

## Methods

### Study design and ethical considerations

This study was conducted as a prospective cohort analysis approved by the Institutional Review Board (IRB No. 15328, approved July 30, 2020) at Penn State College of Medicine. Critically ill adults (≥18 yr) admitted between April 2023 and July 2024, screened via a Modified Early Warning Score-based algorithm and meeting Sepsis-3 criteria within 48 hours of critical illness onset, were included.^14,15^ Written informed consent was obtained from patients with decision-making capacity or from legally authorized representatives before enrollment.

### Study population

The study was performed at a quaternary care, academic center in central Pennsylvania. This 611-bed hospital is a level 1 trauma center, managing a patient population with higher-than-average complexity and acuity, as assessed by multi-specialty case mix index. Critical illness was defined by vasopressor or ventilatory support requirement. Exclusion criteria included comfort-measures-only care, pregnancy, or incomplete data.

### Data collection

Demographics, clinical characteristics, antibiotic usage, and outcomes were extracted from electronic health records (EHR). Structured post-discharge interviews verified follow-up data. AD, AFD, and normalized antibiotic exposure (antibiotic days/days alive, capped at 365) were calculated. Covariates included age, body mass index (BMI), APACHE II score,^16^ and glucocorticosteroid use. Functional status was assessed via Zubrod/ECOG scores. Severity metrics such as SOFA and APACHE II were generated using standardized scoring algorithms. The Zubrod/ECOG Performance Status score, ranging from 0 (fully active) to 5 (death), was collected as a measure of functional status.

### Outcome definitions

Secondary infection was defined as receipt of ≥3 consecutive days of antibiotics following the index sepsis admission. This pragmatic threshold was used to distinguish likely infection-driven treatment from brief empiric coverage. Microbiologic confirmation was not required, reflecting real-world variability in documentation. While no consensus cutoff exists, our approach aligns with prior work^12^ and is intended to identify cases with strong clinical suspicion of infection.

Secondary outcomes included one-year bacteremia, other positive cultures, hospital readmissions, all-cause mortality, and discharge disposition. Multi-drug-resistant organisms (MDROs) were noted. Outcomes were monitored through EHR review and structured phone interviews at 30 days, 3 months, 6 months, and 12 months following study enrollment.

### Statistical analysis

Categorical variables were analyzed via Chi-square or Fisher’s tests, continuous variables via t-tests or nonparametric alternatives. Logistic regression assessed AD and AFD impacts on outcomes, adjusting for confounders. Time-to-event analyses employed Cox proportional hazards models, with death treated as a censoring event at 365 days. Nonparametric tests assessed cumulative infection burden. Adjusted odds ratios (OR) and hazard ratios (HR) with 95% confidence intervals (CI) were reported.

## Results

### Cohort characteristics

A total of 126 critically ill adults were included, of whom 90 (71.4%) met sepsis criteria and 36 (28.6%) were classified as non-septic. Table 1 summarizes demographic and clinical characteristics, demonstrating no significant differences between septic and non-septic groups in mean age, sex distribution, race, body mass index, or severity-of-illness scores. Septic patients had significantly higher leukocyte counts (median 16.7 ×10^3^/µL vs 10.7 ×10^3^/µL, *P* < 0.001) and slightly elevated lactate levels (median 3.0 mg/dL vs 2.6 mg/dL, *P* = 0.04). Although more septic patients required vasopressors at admission, this difference was not statistically significant (48.9% vs 33.3%, *P* = 0.11).

**Table 1.** Patient demographics and outcomes

	Critically Ill, Septic (n = 90)	Critically Ill, Non-Septic (n = 36)	*P*-value
**Age** (mean ± SD)	68.1 ± 16.2	68.1 ± 14.2	1.00
**Female** (n, %)	43 (47.7%)	18 (50.0%)	0.82
**Race** (n, %)			
White	80 (88.9%)	32 (88.9%)	0.31
African American	3 (3.3%)	3 (8.3%)	
Asian, Indian American, or unknown	7 (7.8%)	1 (2.8%)	
**Body Mass Index** (mean ± SD)	34.0 ± 11.2	31.0 ± 9.0	0.12
**Severity of illness**			
APACHE II Score (mean ± SD)	21.2 ± 7.1	19.6 ± 6.6	0.26
SOFA Score (median, IQR)	7.0 (4.8, 10.3)	7.0 (5.0, 9.75)	0.45
Charlson Comorbidity Index (median, IQR)	5.0 (3.0, 7.3)	5.0 (2.25,6.75)	0.56
Patients receiving stress-dosed hydrocortisone 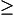 8 h (n, %)	16 (17.8)	2 (5.6)	0.08
Daily hydrocortisone dose (mg) amongst recipients (median, IQR)	175 (128, 196)	142 (133,150)	0.20
Duration of hydrocortisone (days*) amongst recipients (median, IQR)	3 (2.0, 4.7)	2.0 (1.0, 4.0)	0.59
**Laboratory Values**			
Leukocyte Count, x10^3^/µl (median, IQR)	16.7 (12.2, 23.5)	10.7 (7.6,18.3)	**< 0.001**
Lactic Acid (mg/dL) on admission (median, IQR)	3.0 (1.8, 4.9)	2.6 (1.6, 4.5)	**0.04**
Shock (vasopressors and lactate 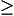 2) on admission (n, %)	44 (48.9%)	12 (33.3%)	0.11
**Short-Term Outcomes**			
Total Proportion of Cohort developing secondary infections, defined by positive culture results (n, %)	12 (13.3%)	9 (25%)	0.12
Mean number of days between enrollment and secondary infection, defined by positive culture results (mean ± SD)	17.9 ± 10.8	13.5 ± 9.5	**0.03**
In-Hospital Mortality Rate (n, %)	16 (17.8%)	5 (13.9%)	0.60
30-Day Mortality Rate (n, %)	19 (21.1%)	5 (13.9%)	0.35
Hospital Length of Stay, days (median, IQR)	9.0 (5, 14.3)	9.0 (5.25,18.8)	0.37
30-day Zubrod/ECOG score (median, IQR)	2.4 (1,4)	1.8 (1,3)	0.08
30-Day Hospital Readmission amongst survivors (n, %)			
90-Day Mortality Rate (n, %)	13 (14.4%)	3 (8.3%)	0.35
Hospital Discharge Disposition, among in-hospital survivors	25 (27.8%)	6 (16.7%)	0.19
---home (n, %)	39 (43.3%)	12 (36.1%)	
---skilled nursing facility (n, %)	27 (30.0%)	15 (41.7%)	
---long-term acute care hospital (LTACH) (n, %)	4 (4.4%)	1 (2.8%)	
**Long-Term Outcomes**			
1-Year All-cause Mortality Rate (n, %)	34 (37.8%)	13 (36.1%)	0.86
1-Year Zubrod/ECOG score (median, IQR)	1.8 (0,4)	1.2 (0,1)	0.24
Place of patient location at 1-year survival			
---home (n, %)	54 (60.0%)	16 (44.4%)	0.11
---skilled nursing facility (n, %)	4(4.4%)	1 (2.7%)	0.67
---long-term acute care hospital (LTACH) (n, %)	1 (1.1%)	0 (0.0%)	0.52

### Infectious etiology

Primary infections among septic patients included gram-positive (30.0%), gram-negative (33.3%), mixed (16.7%), and fungal or viral etiologies (7.7%). Table 2 summarizes infectious etiologies for secondary infections, showing that secondary infections varied broadly, including respiratory, urinary, and soft-tissue infections. Supplementary Table 1 enumerates the infectious etiologies and their corresponding sources.

**Table 2.** Infectious etiology and sources

	Critically Ill, Septic	Critically Ill, Non-Septic	*P*-value
**Etiology of Secondary infections < 30 d**	N = 14	N = 9	0.48
Gram-positive	2 (14.3%)	3 (33.3%)	
Gram-negative	2 (14.3%)	2 (22.2%)	
Fungal	2 (14.3%)	0 (0.0%)	
Viral	1 (7.1%)	1 (11.1%)	
Mixed	4 (28.6%)	3 (33.3%)	
Clinical Diagnosis of Infection only	3 (21.4%)	0 (0.0%)	
**Etiology of Secondary infections 31–365 d**	N = 32	N = 7	0.40
Gram-positive	11 (34.4%)	2 (28.6%)	
Gram-negative	7 (21.9%)	4 (57.1%)	
Fungal	0 (0.0%)	0 (0.0%)	
Viral	2 (6.3%)	0 (0.0%)	
Mixed	3 (9.4%)	1 (14.3%)	
Clinical Diagnosis of Infection only	9 (20.6%)	0 (0.0%)	
**Source of Secondary infections < 30 d**	N = 11	N = 10	0.70
Pneumonia	3 (27.3%)	4 (40.0%)	
Abdominal Infection	1 (9.1%)	1 (10.0%)	
Urinary tract infection	1 (9.1%)	2 (20.0%)	
Skin or Soft Tissue Infection	6 (54.5%)	3 (30.0%)	
Other	0 (0.0%)	0 (0.0%)	
**Source of Secondary infections 31–365 d**	N = 27	N = 6	0.37
Pneumonia	4 (14.8%)	2 (33.3%)	
Abdominal Infection	0 (0.0%)	0 (0.0%)	
Urinary tract infection	12 (44.4%)	1 (16.7%)	
Skin or Soft Tissue Infection	11 (40.7%)	3 (50.0%)	
Other	0 (0.0%)	0 (0.0%)	

### Short-term outcomes

Within 30 days, secondary infections occurred in 13.3% of septic and 25.0% of non-septic patients (*P* = 0.11)(Table 1). Logistic regression did not demonstrate a significant relationship between AFD and 30-day secondary infection (OR 0.97, 95% CI 0.91–1.02, *P* = 0.232). Attempts to model in-hospital mortality were limited by perfect prediction (no deaths occurred in specific AFD categories), a recognized limitation of logistic regression with small sample sizes.^17^

**Figure 1.**
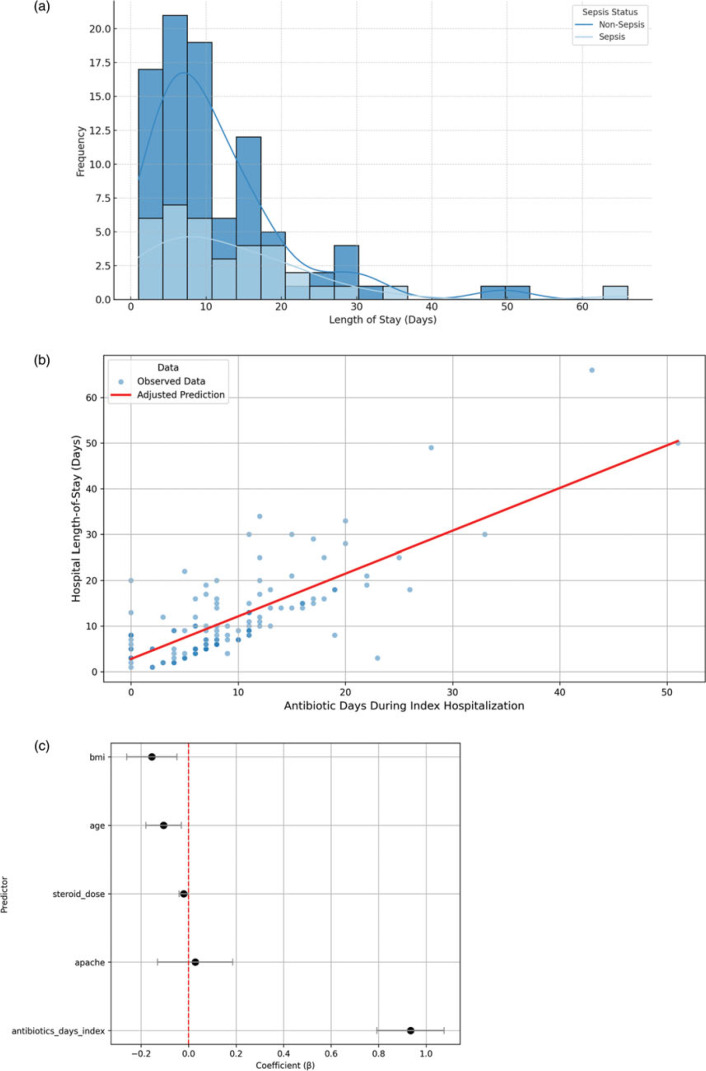
Relationship between the duration of antibiotic use during the index hospitalization and clinical outcomes. (A) Distribution of index hospital length of stay (LOS) among the study cohort. The x-axis represents LOS in days, while the y-axis shows the frequency of patients within each bin. Descriptive statistics include mean LOS (11.9 d), median (9 d), and standard deviation (10.1 d). (B) Adjusted relationship between antibiotic duration (AD) and hospital LOS. Scatterplot of observed hospital LOS by AD during index hospitalization (blue points). The red line represents the adjusted predicted LOS derived from a multivariable linear regression model that included AD, age, APACHE II score, steroid dose, and BMI. Predicted values were calculated while holding all covariates at their mean. Each additional antibiotic day was associated with an approximate 0.93-day increase in hospital LOS (*P* < 0.001). (C) Linear regression model coefficients for predictors of hospital length-of-stay. Each point represents the adjusted regression coefficient (β) for a covariate included in the multivariable linear model of hospital LOS. Horizontal lines represent 95% confidence intervals. A positive coefficient indicates that higher values of the predictor are associated with longer LOS. Notably, AD during index hospitalization was the strongest predictor, with each additional day of antibiotics associated with nearly one additional hospital day. The red dashed line indicates the null effect (β = 0).

Multidrug-resistant organisms (MDROs) were identified in 66.6% (n = 8) of septic patients with secondary infections within 30 days and in 48% (n = 12) occurring between 30 days and one year. In non-septic patients, these rates were 33.3% (n = 6) within 30 days and 60% (n = 6) between 30 days and one year. Median hospital length of stay (LOS) was 9.0 days (IQR 5–14.3) (Table 1 and Figure 1A), significantly associated with AD: in adjusted linear regression, each additional antibiotic day was associated with a 0.93-day increase in hospital stay (*P* < 0.001), independent of age, APACHE II, BMI, and steroid use (Figure 1B and 1C).

### Long-term outcomes

**Figure 2.**
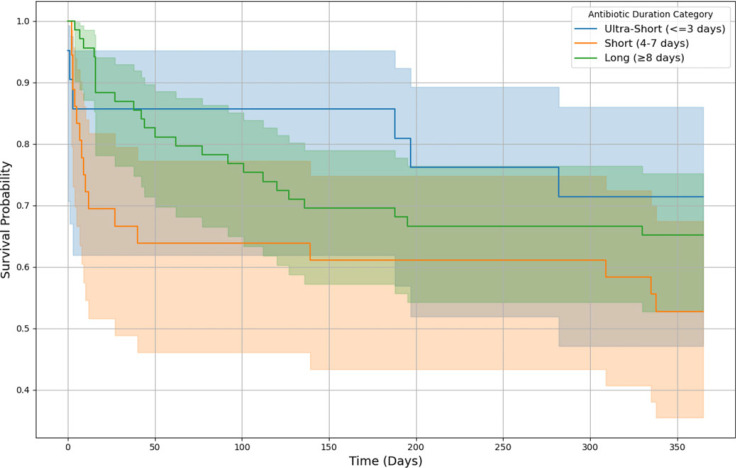
One-year survival by antibiotic duration during index hospitalization. This Kaplan-Meier survival curve illustrates the probability of one-year survival stratified by antibiotic duration (AD) categories during the index hospitalization. The shaded regions represent 95% confidence intervals. Patients who survived beyond one year were censored at 365 days. In the long-duration group (≥8 d), 69 patients were included, with 24 deaths at one year. In the short-duration group (4–7 d), 36 patients were included, of whom 17 died within one year. In the ultra-short group (≤3 d), 21 patients were included, with 6 deaths at one year. There was no significant difference between AD and mortality, after factoring age, APACHE II score, gender and glucocorticosteroid dose as co-variates.

Kaplan-Meier survival curves (Figure 2) showed no significant differences in one-year survival among patients with long (≥8 d), short (4–7 d), or ultra-short (≤3 d) antibiotic courses (log-rank *P* > 0.05). Multivariate analyses confirmed that AD did not significantly predict key one-year outcomes (Table 3).

**Table 3.** Adjusted odds ratios (95% CI) for effect of 28-day antibiotic-free days (AFD) on key long-term outcomes

Outcome	Adjusted OR per day (95% CI)	P-value
Secondary infection (30 d)	0.97 (0.91–1.02)	0.232
Secondary infection (365 d)	1.05 (0.98– 1.12)	0.184
One-year mortality	1.0 (0.95–1.06)	0.992
One-year readmission	0.99 (0.94–1.04)	0.771
One-year bacteremia	1.06 (0.98–1.15)	0.142

### Antibiotic duration and secondary infections

**Figure 3.**
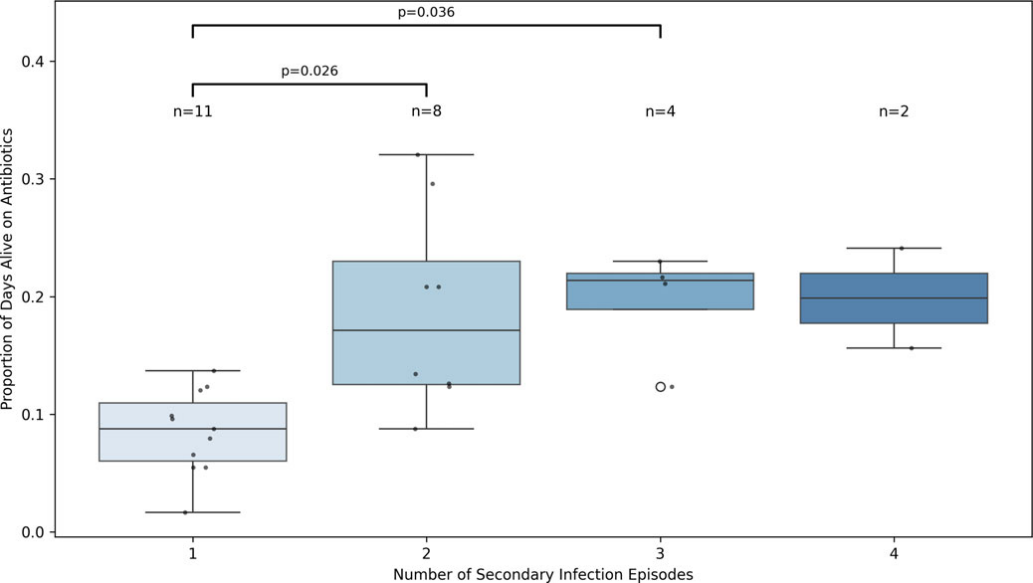
Normalized antibiotic exposure (antibiotic days divided by days alive) stratified by the number of secondary infection episodes within one year. Boxes represent the interquartile range (IQR) with medians; whiskers extend 1.5 times the IQR. Individual patient data points are overlaid as black dots, and the sample size for each group (n) is shown above each box. Open circle signifies and outlier. Group differences were assessed using the Kruskal-Wallis test (H = 13.72, *P* = 0.0033), with pairwise comparisons evaluated by Dunn’s test with Bonferroni correction. Statistically significant comparisons are annotated on the plot. These results diverge from logistic regression analyses using binary secondary infection outcomes, which failed to detect a significant relationship with early antibiotic-free days (AFD). The contrast highlights the added value of continuous, time-normalized exposure metrics in capturing dose-dependent risk gradients for infection recurrence.

As expected, patients with two or more secondary infections demonstrated markedly higher cumulative antibiotic exposure compared to those with only one episode (*P* = 0.026 and *P* = 0.036, respectively; Figure 3). This result persisted even after adjusting for age, APACHE II score, BMI, and corticosteroid dose. These findings contrast with our logistic regression models, which showed no significant association between 28-day AFD and the odds of developing a secondary infection at one year (OR 1.05, 95% CI 0.98–1.12, *P* = 0.184, Table 3). This discrepancy suggests that cumulative exposure to antibiotics may contribute to infection risk in a dose-dependent manner not captured by binary logistic modeling. Importantly, it emphasizes the value of normalized, continuous metrics in detecting graded clinical relationships that may be masked in threshold-based binary outcome analyses.

Cox regression analysis showed a significant relationship AD and *time to* first secondary infection (HR 1.10, 95% CI 1.04–1.15, *P* = 0.001). This suggests that prolonged antibiotic use is associated with earlier onset of secondary infections, in addition to elevating cumulative infection risk.

### Subgroup analyses

Sepsis status was not independently associated with short- or long-term mortality, readmission, or bacteremia risk. While an initial unadjusted analysis suggested an inverse association between 28-day AFD and subsequent bloodstream infections, this relationship was attenuated and lost statistical significance after adjustment. Covariates may reflect underlying patient frailty or immune status and could confound infection risk. Importantly, normalized antibiotic exposure remained independently associated with an increased risk of secondary infections (defined by positive cultures) at one year, suggesting that prolonged antibiotic exposure may drive microbial dysbiosis or antimicrobial resistance. Notably, the microbiologic etiology of the index infection—including gram-positive, gram-negative, fungal, or viral sources—did not significantly influence either AD or the risk of subsequent infections.

## Discussion

In this prospective cohort study of critically ill adults, we found that AD during the index hospitalization was significantly associated with prolonged hospital LOS and increased likelihood of developing secondary infections. However, the strength of these associations varied based on the modeling approach used. While logistic regression analyses using binary AFD measures did not consistently yield statistically significant results for secondary infections, time-to-event models and normalized exposure analyses did reveal significant dose-dependent relationships.

These findings suggest that modeling approaches which account for timing and intensity of antibiotic exposure—such as Cox proportional hazards models or normalized antibiotic duration (ie, proportion of days alive on antibiotics)—may be more sensitive to uncovering the adverse effects of prolonged antibiotic use than simpler binary measures. The observed attenuation of associations in traditional regression models underscores the potential limitations of relying solely on cumulative antibiotic-free days, which do not capture nuanced exposure patterns or account for survival time.

Moreover, the divergence in findings between models highlights the importance of method selection when evaluating longitudinal antibiotic effects. Whereas logistic models assess the probability of an outcome without accounting for timing, survival analysis incorporates both the occurrence and timing of events. Similarly, normalized AD controls for variation in survival time, offering a more accurate assessment of relative antibiotic exposure, especially among patients who may have died early or were discharged quickly.

Thus, our study not only identifies a clinically meaningful relationship between prolonged antibiotic use and downstream infectious risks but also emphasizes the importance of aligning exposure metrics with appropriate analytical frameworks. Antibiotic stewardship efforts in critical care should consider adopting time- and exposure-adjusted metrics when evaluating the safety and efficacy of antibiotic protocols.

Prior studies, including the BALANCE and SAFE trials, support shorter antibiotic regimens for bloodstream infections and community-acquired pneumonia, demonstrating non-inferiority compared to longer courses.^18–20^ Meta-analyses of community-acquired pneumonia^19,21–24^ and other infections^25–30^ further support that antibiotic regimens of seven days or fewer are safe and effective for many clinical scenarios. More targeted investigations focused on pathogens such as *Staphylococcus aureus*, vancomycin-resistant Enterococci, and *Pseudomonas aeruginosa* corroborate that reduced durations do not necessarily worsen survival or readmission rates.^31–34^ Moreover, some studies have demonstrated that prolonged AD are actually harmful^35^ and could increase mortality.^30^ Our findings align with these studies, underscoring the benefits of abbreviated AD across various infectious etiologies in critically ill populations.

The limitations of our study include its single-center design, relatively small sample size, and reliance on clinical rather than microbiologically confirmed definitions of secondary infections. These factors may limit the generalizability and precision of our results. Furthermore, our definition of secondary infection (≥3 d of antibiotics) is a pragmatic proxy for a treated infection, based on real-word practice of empiric antibiotic coverage until infection is ruled out. We recognize that without standardized microbiological criteria or adjudication, some patients on prolonged empiric therapy may be misclassified, and conversely short-course infections might be missed. Nonetheless, the robust associations observed between prolonged antibiotic use and increased hospital length-of-stay, coupled with higher cumulative infection burden, provide important clinical insights.

## Conclusion

In this prospective cohort study of critically ill adults, prolonged AD during the index hospitalization was significantly associated with longer hospital stays and earlier onset of secondary infections, but did not improve one-year survival or readmission rates. Importantly, while traditional logistic regression using 28-day AFD failed to detect a significant association with secondary infections, time-to-event and normalized exposure models revealed strong dose-dependent relationships. These findings suggest that cumulative and time-adjusted antibiotic exposure metrics may more sensitively capture the risks of prolonged antibiotic use, including microbial dysbiosis and recurrent infections. Our results underscore the importance of antibiotic stewardship in critical care, emphasizing that shorter, targeted antibiotic courses may minimize unintended harm without compromising long-term outcomes.

## Supporting information

10.1017/ash.2025.10054.sm001 Burrows et al. supplementary material Burrows et al. supplementary material

## Data Availability

Data are available from the authors on reasonable request.

## Supplementary material

The supplementary material for this article can be found at https://doi.org/10.1017/ash.2025.10054.
