# Supplementary material for: Association between in-hospital antibiotic use and long-term outcomes in critically ill patients
Source: Antimicrob Steward Healthc Epidemiol. 2025 Jun 23;5(1):e135. doi: 10.1017/ash.2025.10054 (PMC12188279; doi:10.1017/ash.2025.10054)
Supplement: Burrows et al. supplementary material [file S2732494X25100545sup001.docx]

**Supplementary Materials**

**Supplementary Table 1.** Microbial etiology and infectious source within the study cohort

| **Name of Organism cultured** | **Blood** | **Urine** | **Respiratory** | **Abdomen** | **Soft tissue/skin** |
| --- | --- | --- | --- | --- | --- |
| *Actinomyces odontolyticus* |  |  |  | 1 |  |
| *Aerococcus urinae* |  | 1 |  |  |  |
| *Anaerobic gram-positive rods (unspeciated)* |  |  |  | 1 |  |
| *Bacteroides fragilis* | 1 |  |  |  |  |
| *Bacteroides pyogenes* |  |  |  | 1 |  |
| Bacteroides thetaiotaomicron |  |  |  | 1 |  |
| Beta streptococcus, group B | 4 |  | 1 |  |  |
| Beta streptococcus, group G | 1 |  |  |  |  |
| Beta streptococcus, group A | 3 |  |  |  | 1 |
| *Bifidobacterium* |  |  |  | 1 |  |
| *Candida albicans* |  |  |  | 1 |  |
| *Candida (unspeciated)* |  |  | 1 |  |  |
| *Candida glabrata* | 3 | 1 | 1 | 1 |  |
| *Candida lusitaniae* |  |  | 1 | 1 |  |
| *Clostridium clostridiforme* |  |  |  | 1 |  |
| *Clostridium difficile* |  |  |  | 1 |  |
| *Clostridium perfringens* |  |  |  | 2 |  |
| *Corynebacterium striatum* | 1 |  |  |  |  |
| SARS-CoV-2 |  |  | 5 |  |  |
| *Enterobacter cloacae* | 1 |  | 1 | 1 |  |
| *Enterococcus* (unspeciated) | 1 |  |  |  |  |
| *Enterococcus faecalis* |  | 2 |  | 1 |  |
| *Enterococcus faecium* |  | 2 |  | 1 |  |
| *Escherichia coli* | 8 | 6 | 2 | 5 | 1 |
| *Fusobacterium nucleatum* | 1 |  |  |  |  |
| *Gemella morbillorum* | 1 |  |  |  |  |
| Gram-negative rods (unspeciated) |  |  | 2 |  |  |
| *Haemophilus influenza* |  |  | 2 |  |  |
| Influenza A |  |  | 2 |  |  |
| *Klebsiella oxytoca* | 2 | 1 | 1 |  |  |
| *Klebsiella pneumoniae* | 4 | 4 |  | 1 |  |
| *Staphylococcus aureus* | 4 |  | 7 |  | 5 |
| *Morganella morganii* | 1 |  |  | 1 | 1 |
| *Prevotella intermedia* | 1 |  |  |  | 1 |
| *Proteus mirabilis* | 5 | 6 |  |  |  |
| *Proteus vulgaris* | 1 |  |  |  |  |
| *Pseudomonas aeruginosa* |  | 1 | 2 |  |  |
| Rhinovirus/enterovirus |  |  | 1 |  |  |
| *Rhodotorula* |  |  | 1 |  |  |
| *Serratia marcescens* |  | 1 |  |  |  |
| *Staphylococcus epidermidis* (two positive blood cultures) | 2 |  |  |  |  |
| *Staphylococcus lugdenensis* | 2 |  | 1 |  |  |
| *Streptococcus anginosus* | 1 |  |  |  | 1 |
| *Streptococcus mutans* | 1 |  |  |  |  |
| *Streptococcus viridans* |  |  | 1 |  |  |

* If multiple microbes were isolated from a patient's microbial cultures, each microbe is listed individually.


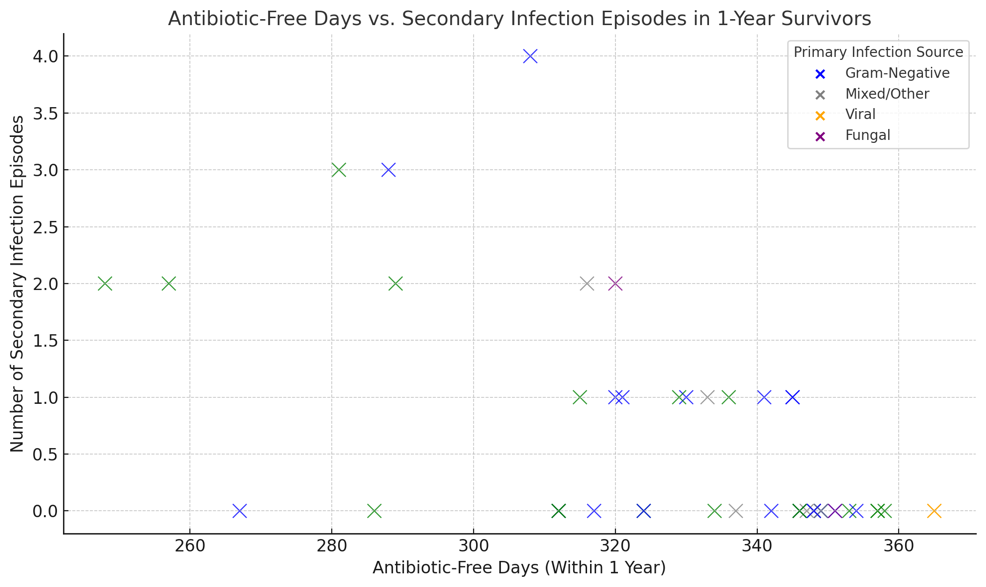


**Supplementary Figure 1. Relationship between antibiotic-free days and secondary infection, in 1-year survivors**, stratified by primary infection source. Each “X” represents one patient; n=49.
